# Double-balloon endoscopy-assisted peroral pancreatoscopy using a novel slim cholangiopancreatoscope for the diagnosis of pancreatic duct stricture in surgically altered anatomy

**DOI:** 10.1055/a-2615-5923

**Published:** 2025-07-02

**Authors:** Arata Oka, Haruka Toyonaga, Takuya Takayama, Tatsuya Nakagawa, Masahiro Orino, Hironao Matsumoto, Masaaki Shimatani

**Affiliations:** 1 Department of Gastroenterology and Hepatology, Kansai Medical University Medical Center, Moriguchi, Japan

Peroral pancreatoscopy (POPS) enables direct visualization of intraductal lesions, targeted biopsy, and therapeutic interventions, playing an important role in evaluating pancreatic ductal lesions 1 2 . However, in patients with surgically altered anatomy, POPS is technically challenging owing to the need for scope exchange, difficulties in advancing into the pancreatic duct, and limited maneuverability.

A newly developed slim cholangiopancreatoscope (eyeMAX 9 Fr; Micro-Tech, Nanjing, China) has a thin outer diameter that can pass through the working channel of a balloon-assisted endoscope 3 4 5 . Herein, we report the utility of double-balloon endoscopy-assisted peroral pancreatoscopy (DB-POPS) using a novel slim cholangiopancreatoscope to directly visualize and biopsy a pancreatic tail duct stricture in a patient with surgically altered anatomy ( Video 1 ).

Double-balloon endoscopy-assisted peroral pancreatoscopy using a novel slim cholangiopancreatoscope enhanced diagnostic precision in evaluating pancreatic ductal lesions in a patient with surgically altered anatomy by enabling direct, stable, and simplified visualization and biopsy.Video 1

A man in his 70s with a history of Billroth-II reconstruction was referred due to recurrent pancreatitis. Imaging findings revealed a localized stricture in the pancreatic tail duct with upstream cystic dilatation ( Fig. 1 ). Although endoscopic ultrasound revealed no distinct hypoechoic mass suggestive of advanced pancreatic cancer, a localized stricture of the main pancreatic duct was observed in the pancreatic tail ( Fig. 2 ).

**Fig. 1:**
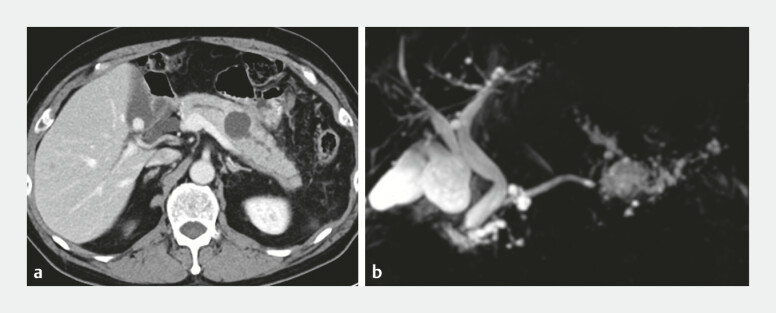
Imaging studies performed to investigate the cause of recurrent pancreatitis revealed localized stricture of the main pancreatic duct in the pancreatic tail and cystic dilatation of the proximal pancreatic duct. No solid mass suggestive of advanced pancreatic cancer was identified. **a** Contrast-enhanced computed tomography. **b** Magnetic resonance cholangiopancreatography.

**Fig. 2:**
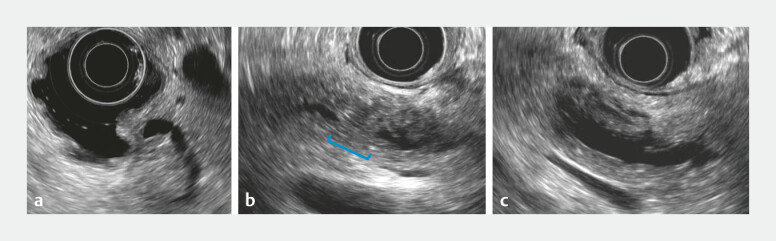
Endoscopic ultrasound (EUS) images. **a** EUS revealed no obvious obstructive lesion at the duodenal papilla. **b, c** Although no distinct hypoechoic mass suggestive of advanced pancreatic cancer was identified, a localized stricture of the main pancreatic duct was observed in the pancreatic tail. The proximal pancreatic duct was cystically dilated, and thickening of the ductal wall was noted.

Double-balloon endoscopy-assisted endoscopic retrograde cholangiopancreatography confirmed pancreatic tail duct stricture and upstream dilatation ( Fig. 3 ). Brush cytology, fluoroscopy-guided biopsy at the stricture, and serial pancreatic juice aspiration cytology revealed no malignancy. Thus, the novel slim cholangiopancreatoscope was introduced through the working channel of the balloon-assisted endoscope for direct observation and biopsy. DB-POPS visualized the tight stricture, with irregular protrusions and abnormal vessels beyond it. Targeted biopsies from the lesion and repeated cytology demonstrated atypical cells, raising suspicion of malignancy ( Fig. 4 ).

**Fig. 3:**
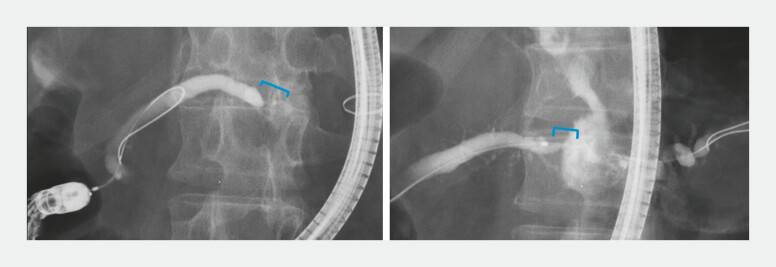
Double-balloon endoscopy-assisted endoscopic retrograde cholangiopancreatography revealed a localized stricture of the main pancreatic duct in the pancreatic tail, with dilation of the upstream pancreatic duct.

**Fig. 4:**
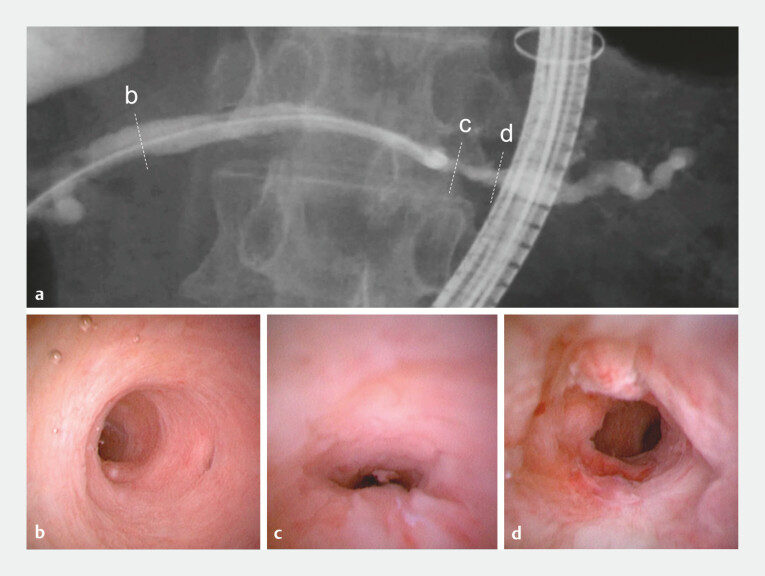
Double-balloon (DB) endoscopy-assisted endoscopic retrograde cholangiopancreatography ( **a** ) and DB-assisted peroral pancreatoscopy (POPS) using a novel slim cholangiopancreatoscope (eyeMAX 9 Fr; Micro-Tech, Nanjing, China) ( **b–d** ). Direct visualization revealed normal pancreatic duct mucosa in the pancreatic head/body ( **b** ), and a tight stricture in the pancreatic tail ( **c** ), beyond which irregular protrusions accompanied by tortuous and dilated abnormal vessels were observed ( **d** ).

Considering the recurrent pancreatitis caused by the pancreatic duct stricture, along with DB-POPS and pathological findings, distal pancreatectomy was performed due to suspicion of early-stage pancreatic cancer.

DB-POPS utilizing a novel slim cholangiopancreatoscope enhanced the diagnostic precision when evaluating pancreatic ductal lesions in a patient with surgically altered anatomy by enabling direct, stable, and simplified visualization and biopsy.

Endoscopy_UCTN_Code_TTT_1AR_2AD

## Notes

Endoscopy E-Videos https://eref.thieme.de/e-videos
*E-Videos* is an open access online section of the journal *Endoscopy* , reporting on interesting cases and new techniques in gastroenterological endoscopy. All papers include a high-quality video and are published with a Creative Commons CC-BY license. Endoscopy E-Videos qualify for HINARI discounts and waivers and eligibility is automatically checked during the submission process. We grant 100% waivers to articles whose corresponding authors are based in Group A countries and 50% waivers to those who are based in Group B countries as classified by Research4Life (see: https://www.research4life.org/access/eligibility/ ). This section has its own submission website at https://mc.manuscriptcentral.com/e-videos .
